# Herbal Melanin Inhibits Colorectal Cancer Cell Motility, Invasiveness, and Epithelial–Mesenchymal Transition, Associated with u-PAR Downregulation Through JNK and ERK Pathways

**DOI:** 10.3390/cimb48040353

**Published:** 2026-03-27

**Authors:** Maha-Hamadien Abdulla, Ahmad Al Zahrani, Mansoor-Ali Vaali-Mohammed, Sabine Matou-Nasri, Abdullah O. Al Obeed, Thamer Bin Traiki, Noura S. Alhassan

**Affiliations:** 1 Department of Surgery, College of Medicine, King Saud University, Riyadh 11472, Saudi Arabia; vmansoorali@ksu.edu.sa (M.-A.V.-M.); ab.alobaid99@gmail.com (A.O.A.O.); ttraiki@ksu.edu.sa (T.B.T.);; 2 Department of General Surgery, College of Medicine, Majmaah University, Majmaah 15341, Saudi Arabia; ama.565@hotmail.com; 3 Blood and Cancer Research Department, King Abdullah International Medical Research Center, King Saud bin Abdulaziz University for Health Sciences, Ministry of National Guard-Health Affairs, Riyadh 11481, Saudi Arabia; matouepnasrisa@mngha.med.sa; 4 Biosciences Department, Faculty of the School of Systems Biology, George Mason University, Manassas, VA 20110, USA

**Keywords:** colorectal cancer, herbal melanin, urokinase-type plasminogen activator receptor, epithelial–mesenchymal transition

## Abstract

Herbal melanin (HM), previously reported for its antiproliferative and pro-apoptotic properties, has garnered interest as a promising anti-colorectal cancer drug. However, HM’s biological effects and underlying molecular mechanisms and the related signaling pathways in colorectal cancer (CRC) cell motility are poorly investigated. To evaluate the impact of various concentrations (50, 100, and 200 μg/mL) of HM on cell migration, invasion, and tumorigenicity on human HT29 and SW620 CRC cell lines, a real-time cell analyzer instrument and colony formation assays were employed, respectively. An angiogenesis-related protein array was also used, and the levels of protein expression contributing to colony formation and extracellular proteolysis-driven cell migration and invasion, such as E-cadherin, N-cadherin and urokinase-type plasminogen activator receptor (uPAR), were monitored using Western blotting and RT-qPCR technologies. HM significantly decreased CRC cell motility, invasiveness, and formation of colonies, associated with E-cadherin upregulation and N-cadherin downregulation. In addition, HM specifically inhibited uPAR expression levels, which were also decreased by the pharmacological mitogen-activated protein kinase (MAPK) kinase (MEK) inhibitor UO126 and Jun N-terminal kinase (JNK) inhibitor SP600125, in both CRC cell lines, including metastatic CRC (mCRC) SW620 cell line. Addition of HM to cells pretreated with JNK and MEK inhibitors attenuated the blockade of JNK and ERK phosphorylation and alleviated HM-downregulated uPAR expression and HM-inhibited mCRC cell migration. In conclusion, our in vitro studies demonstrate that HM exhibits an inhibitory effect on CRC migration and invasiveness, associated with uPAR downregulation through JNK and ERK pathways.

## 1. Introduction

Colorectal cancer (CRC) is the fourth most common cause of cancer-related deaths globally and is a potentially fatal disease of the digestive tract [1]. Patient deaths are primarily caused by the relentless growth of CRC cells and their infiltration into the adjacent tissue or to distant organs, facilitated by metastasis, a multi-step process involving cancer cell motility and invasion. It has been reported that metastases occur in 25% of patients at the time of their initial diagnosis, and a comparable percentage of patients experience metastases as their disease progresses [2,3]. Excision is the treatment of choice for colon wall tumors (stages I and II), and adjuvant chemotherapy combined with surgery is the treatment of choice for about 73% of CRCs (stage III) with lymph node metastases [4]. Chemotherapy has increased patient survival rates more recently, but tumors that have spread via metastasis to distant locations are frequently incurable [5]. Therefore, in order to identify possible targets for early CRC diagnosis, recurrence, and metastasis, it is imperative that the molecular mechanisms underlying the occurrence, development, and progression of CRC be thoroughly investigated.

Numerous tissue-remodeling-related proteins, such as the cell surface urokinase-type plasminogen activator receptor (uPAR), are involved in the growth and progression of tumors [6,7,8]. Tissue remodeling, inflammation, and numerous cancer types—brain cancer [9], gastric cancer [10,11], prostate cancer [12,13], bladder cancer [14,15], melanoma [16], breast cancer [17,18], and colon cancer [19]—all show increased expression levels of the *uPAR* gene. Furthermore, invasive tumors also exhibit high expression of uPA [20]. A plethora of signals are mediated by uPAR and result in the stimulation of tumor-related glycolysis, tumor cell proliferation, tumor microenvironment and angiogenesis (i.e., new blood vessel formation from pre-existent ones), and metastasis, contributing to tumor angiogenesis and, subsequently, tumor progression [21,22,23]. The interaction between uPA and uPAR has the potential to stimulate oncogene expression and cell mitosis, ultimately resulting in tumor growth [24]. CRISPR/Cas9-system-mediated *uPAR* gene knockout in cancer cells inhibits the growth, migration, and invasion of cancer cells [25]. In CRC, *PIK3CA* and *KRAS* mutations delay the development of cancer hallmarks but only when the uPAR expression level on the cell surface is reduced [26]. Targeting uPAR has become an emerging anticancer therapeutic strategy, leading to the development of novel anticancer compounds, including peptide inhibitors, small inhibitors, bioengineered drugs, and natural products [27,28].

Melanin is a group of tyrosine-based natural pigments composed of indole polymers generically named allomelanin, produced in plants, and eumelanin, pheomelanin, and neuromelanin, produced in animals and microorganisms [29]. Animal melanin is biosynthesized in melanosomes, specialized organelles in skin cells and hair follicles called melanocytes [30]. Melanins, particularly herbal melanin (HM), a dark pigment obtained from the seed coat of the Mediterranean plant *Nigella sativa* L., exhibit numerous protective activities, including antioxidant, anti-inflammatory, immuno-modulatory, hypoglycemic, and antitumor activities [31]. From cultured human monocytes and peripheral blood mononuclear cells (PBMCs), HM has been shown to modulate the cytokine production of vascular endothelial growth factor, a well-known pro-angiogenic growth factor, tumor necrosis factor-alpha, and interleukin-6 [32]. Additionally, various in vitro anticancer activities of HM have been reported against hematological malignancies such as human acute monocytic leukemia [33] and solid tumors such as breast and colorectal cancer through a decrease in cell growth by apoptosis induction and modulation of mitogen-activated protein kinase (MAPK) signaling through its main receptor toll-like receptor (TLR4) [34,35]. Thus, numerous in vitro investigations underscore the promising potential of HM in the oncology field as an alternative natural systemic chemotherapeutic agent with fewer side effects as a complementary drug to conventional therapies and as a reliable nanotheranostic tool [31,32,33,34,35,36]. However, the potential anti-metastatic properties of HM have not yet been investigated. Therefore, here, we studied the potential HM effect on other angiogenesis-related proteins, such as uPAR, using CRC cell lines, HT29 as a model of colorectal adenocarcinoma and SW620 as a model of metastatic CRC (mCRC). The main cellular events responsible for cancer progression and tumorigenesis were explored, as well as the associated signaling pathways.

## 2. Materials and Methods

### 2.1. Cell Culture and Treatment

The human colorectal adenocarcinoma cell line HT29 (#HTB-38, harboring mutations, including BRAF-heterozygous-c.1799T>A; PIK3CA-heterozygous-c.1345C>A; TP53-homozygous-c.818G>A) and mCRC cell line SW620 (#CCL-227, harboring mutations, including KRAS-homozygous-c.35G>T; TP53-heterozygous-c.818G>A; and TP53-heterozygous-c.925C>T), purchased from American Type Culture Collection (ATCC, Manassas, VA, USA), were cultured in 10% heat-inactivated fetal bovine serum (HI-FBS, Thermo Fisher Scientific, Waltham, MA, USA), 100 μg/mL streptomycin, 100 IU/mL penicillin, and 2 mmol/L L-glutamine added to Dulbecco’s modified Eagle medium (DMEM, Invitrogen, Thermo Fisher Scientific). The cells were cultured in a 5% CO_2_ incubator at 37 °C with a humidified atmosphere. Using enzymatic digestion with 0.05% trypsin/0.02% ethylenediaminetetraacetic acid (EDTA) and splitting the cells into 1:2 or 1:3 ratios, the cells were passaged every 2 to 3 days until confluence. Throughout the investigation, the cells were employed between passages 5 and 9 [37].

The same batch of HM was prepared and characterized as previously described in [32,38]. Briefly, HM, purchased from a local public herbarium in Riyadh (Saudi Arabia), was extracted from *Nigella sativa* L. seed coats by alkaline solubilization and acid aggregation, purified by centrifugation and filtration, and then vacuum-dried. A 1 g/L solution of lyophilized HM extract was dissolved in 1 N sodium hydroxide and then adjusted to pH 7.0 and filtered through sterile 0.22 μm filters. A stock solution of HM (10 mg/mL) was prepared in sterile water, and no endotoxin contamination was detected. Melanins are negatively charged, hydrophobic, dark, high-molecular-weight irregular biopolymers composed of polymerized phenolic and/or indolic compounds. The melanin nature of the extract was verified via the standard analytical techniques: electron spin resonance (ESR), infrared (IR), ultraviolet–visible (UV-VIS), nuclear magnetic resonance (NMR), X-ray diffraction (XRD), fluorescence, solubility studies, amino acid composition, and elemental analysis. The elemental analysis of carbon, hydrogen, and nitrogen gave (*wt*/*wt* percent) ratios of 50.29, 5.77, and 4.4, respectively, confirming the close similar characteristics to eumelanins [32].

HT29 and SW620 cells were exposed to various concentrations of HM, with or without the pharmacological Jun N-terminal kinase (JNK) inhibitor SP600125 (#S5567, Sigma-Aldrich, Saint Louis, MO, USA) or the pharmacological MAPK kinase (MEK) inhibitor UO126 (#662005, Sigma-Aldrich). From pilot studies, tested at serial concentrations (2.5 μM, 5 μM, 10 μM, and 20 μM) of pharmacological inhibitors, the optimal concentration of SP600125 and UO126 was found to be 10 μM.

### 2.2. Clonogenic Assay

HT29 and metastatic SW620 CRC cells (500 per well) were cultured in 6-well plates. The next day, the cells were treated with HM (50, 100, and 200 µg/mL) for 24 h of incubation. For 14 days, the frequency of medium replacement was every other day, the duration required for the formation of colonies. After that, the cells were thoroughly rinsed with phosphate-buffered saline (PBS) and fixed with 70% ethanol, and the colonies were stained with 0.5% crystal violet [37].

### 2.3. Cell Migration and Invasion Assays Using xCELLigence Real-Time Cell Analysis

To evaluate the HM effect on the ability of CRC cell motility and invasiveness over time, an xCELLigence Real-time Cell Analyzer Dual-Purpose (RTCA-DP) instrument was employed as advised by the manufacturer (ACEA Biosciences Inc., Santa Clara, CA, USA). For the cell motility assessment, a 16-well Cellular Invasion/Migration plate (#CIM-16, Roche Diagnostics GmbH, Mannheim, Germany) was used. The CIM-16 plate contained two chambers; the lower chamber was filled with complete medium (supplemented with 10% HI-FBS), and the upper chamber was filled with serum-free medium to hydrate the membrane. The CIM-16 plates were placed on the RTCA-DP machine station after the incubation period but before the cells were added. The baseline measurement step was carried out against the background signal produced by cell-free medium. Following a count of HT29 and SW620 cells, 12,000 cells were seeded into the upper chamber’s wells. The cell migration was calculated using a gold microelectrode plated on the underside of the upper-chamber membrane to record changes in electrical impedance. The number of cells migrating to the membrane’s underside increased in direct proportion to the increase in impedance. The related RTCA software 1.2.1 (ACEA Biosciences Inc.) was utilized for plotting and calculating the cell index values. After that, for 200 h, the cell index curves were recorded every 15 min to evaluate cell migration. Using the xCELLigence system and CIM-16 plate, the same protocol was applied for the cell invasion assay; however, the upper chamber was coated with Matrigel^™^, a reconstituted basement membrane (#356234, Becton Dickinson Biosciences, Franklin Lakes, NJ, USA) [37].

### 2.4. Angiogenesis-Related Protein Array

CRC cell lysates were subjected to a Human Angiogenesis Antibody Array kit (#ab193655, Abcam, Cambridge, MA, USA) as per the recommendations of the manufacturer. Briefly, HT29 and metastatic SW620 CRC cells were lysed, and the amount of proteins was estimated using a bicinchoninic (BCA) protein assay on a SmartSpec^™^ Plus spectrometer (Bio-Rad Laboratories, Hercules, CA, USA). The provided membranes conjugated with proteins were saturated with blocking buffer (#ab193655, Abcam) for 30 min at room temperature (RT). Following incubation, a volume equivalent to the protein sample was added to half of the blocking buffer, and the mixture was then incubated for 1.5–5 h at RT. After another 1.5–2-h at RT, the membranes were washed and conjugated with a biotinylated antibody cocktail. After that, the membranes were washed and incubated for 2 h at RT with streptavidin conjugated with horseradish peroxidase (HRP). The membranes were then incubated with detection buffer (#ab193655, Abcam), and a luminescent image analyzer (Amersham^™^ Imager 680, Agilent Technologies, Santa Clara, CA, USA) was used to detect the chemiluminescence signals (#ab193655, Abcam). A C-DiGit^™^ Blot Scanner (LI-COR Biosciences, Lincoln, NE, USA) was used to quantify the signal intensity, and the LI-COR Image Studio^™^ software (version 6.0) was used to take and process the images. Data quantification was performed using SigmaPlot (version 16.0).

### 2.5. Whole-Cell Lysates and Western Blotting

Using a Bradford Protein assay kit (Bio-Rad Laboratories), whole-cell lysates were prepared in radioimmunoprecipitation assay (RIPA) lysis buffer (Boston Bio Products, Ashland, MA, USA). The protein concentration was then measured. Using the Bio-Rad Trans-Blot^®^ Turbo^™^ transfer system, equal quantities of cell lysate proteins were loaded and electrophoresed on 4–20% Mini-Protean TGX precast gels (Bio-Rad Laboratories) before being transferred to a nitrocellulose transfer membrane (0.22 μm). Bio-Rad blotting-grade blocker, 5% skimmed milk, and PBS containing 0.1% Tween-20 (PBST) were used to block the membranes for 1 h at RT. The membranes were then washed twice with PBST. The following primary antibodies were used to probe the membranes for an entire night at 4 °C [37]: they were directed against E-cadherin (#ab231303, 1:1000 dilution, Abcam), N-cadherin (#ab211126, 1:1000 dilution, Abcam), uPAR (#MA5-23853, 1:1000 dilution, Invitrogen, Waltham, MA, USA), phospho(p)JNK (#sc-517383, 1:1000 dilution, Santa Cruz biotechnology, Dallas, TX, USA), phospho-extracellular signal-regulated kinase (pERK, #sc-377400, 1:1000 dilution, Santa Cruz Biotechnology), and β-actin (#sc-47778, 1:1000 dilution, Santa Cruz Biotechnology). Subsequently, immunoreactivity was visualized by chemiluminescence detection using Clarity Western ECL Substrate (#1705061, Bio-Rad Laboratories) after exposing the probed membranes to goat anti-mouse IgG (H+L) secondary antibodies (#31430; 1:20,000 dilution, Invitrogen). Images were captured with a LI-COR C-DiGit^™^ Blot Scanner and analyzed using the LI-COR Image Studio^™^ software.

### 2.6. RNA Extraction and Reverse-Transcription Polymerase Chain Reaction (RT-PCR)

After harvesting non-treated and treated HT29 and metastatic SW620 CRC cells, whole RNA was extracted using an Ambion^®^ RecoverAll^™^ Total Nucleic Acid Isolation Kit (#AM1975, Invitrogen). Using an ultraviolet spectrophotometer, the A260/280 ratio (1.8–2.0) was measured to determine the purity of total RNA. RNAse-free DNA enzyme (#AM2224, Ambion^®^, Austin, TX, USA) was used to treat RNA samples in order to eliminate genomic DNA contamination. A set of commercially available High-Capacity complementary DNA (cDNA) Reverse Transcription kits (#4368814, Applied Biosystems, Thermo Fisher Scientific) were used to carry out the reverse transcription reaction. Using random hexamer primers, 2 μg of total RNA was converted to cDNA. The mixture was prepared as follows: 10 min at 25 °C, 2 h at 37 °C, 5 min at 85 °C on a PCR thermocycler Gene (Applied Biosystems), and maintenance at 4 °C. A final concentration of 5 ng/μL of diluted cDNA served as a template for the PCR preparation process. PCR samples were generated using an iScript One-step RT-PCR kit with SYBR^®^ Green (#1725120, Bio-Rad Laboratories) and visualized on a LightCycler^®^ real-time PCR system (Roche Diagnostics, Basel, Switzerland) [37]. To determine the relative quantification of PCR products, the 2^−^^ΔΔCT^ method was utilized. The levels of expression of *E-cadherin*, *N-cadherin*, and *uPAR* genes were measured relative to the expression level of glyceraldehyde 3-phosphate dehydrogenase (*GAPDH*) gene. Table 1 lists the primer sequences that were used.

### 2.7. Statistical Analysis

The three separate experiments’ mean and standard deviation (SD) are displayed as the results. A one-way ANOVA statistical test was used to examine the difference between the control and treated groups, and Student’s *t*-test was utilized for statistical analysis. The *p*-values that were below 0.05 were deemed statistically significant.

## 3. Results

### 3.1. HM Decreases CRC Cell Motility and Invasiveness and Inhibits Colony Formation Associated with Differential Effects on Epithelial–Mesenchymal Transition (EMT) Marker Expression

**Figure 1:**
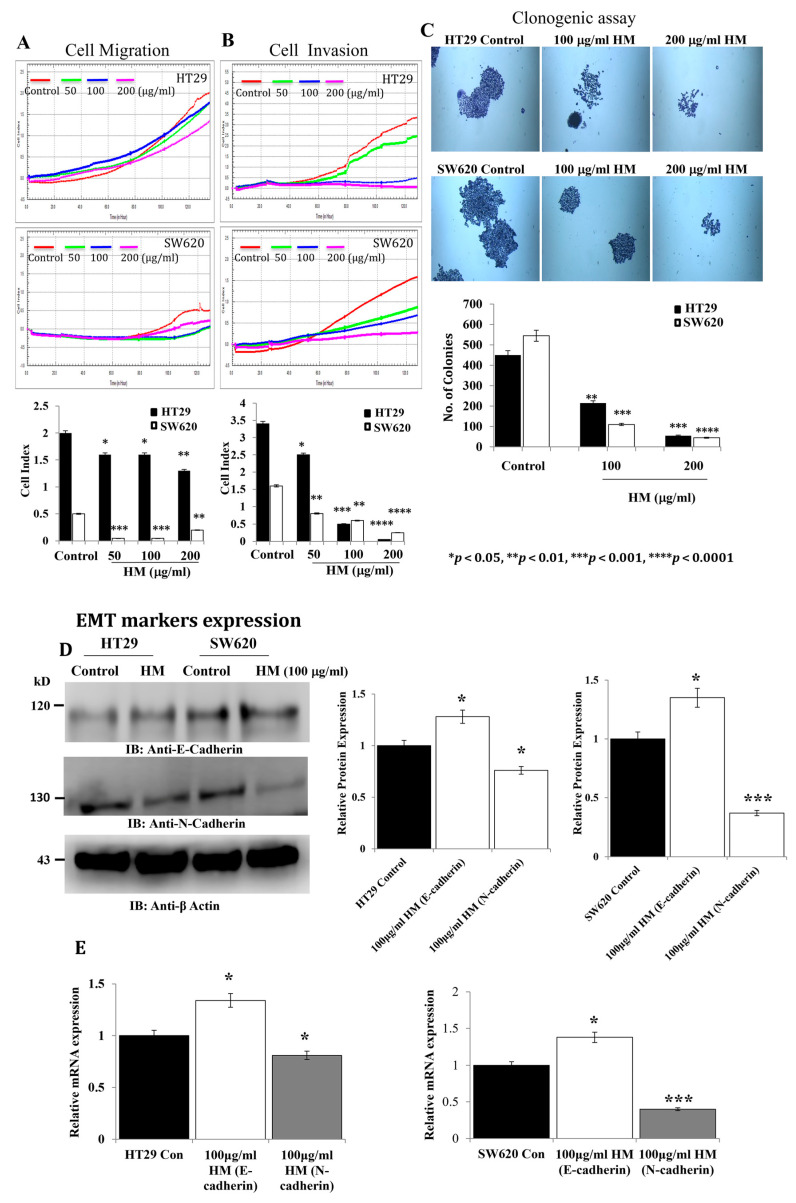
**HM inhibits colony formation, cell motility, and invasiveness in HT29 and SW620 cell lines.** (**A**) In order to promote colony formation, HT29 and SW620 cells were incubated for 10–12 days at 37 °C alongside untreated HT29 and SW620 cells. Using a light microscope, the number of colonies was counted after applying crystal violet staining. An xCELLigence RTCA-DP system was used to monitor real-time CRC cell migration (**B**) and invasion (**C**). (**D**) In HT29 and SW620 cell lines, HM increases the expression of E-cadherin and decreases N-cadherin. Whole-cell lysates were subjected to immunoblotting using the designated antibodies directed against E-cadherin and N-cadherin. The bar graph shows the relative level of protein expression in relation to the loading control, β-actin. (**E**) A bar graph illustrating the relative levels of *N-cadherin* and *E-cadherin* mRNA expression in relation to *GAPDH* mRNA, the internal control. Results from three separate experiments (*n* = 3) are displayed in bar graphs as mean ± SD. When reporting * *p* < 0.05, ** *p* < 0.01, *** *p* < 0.001, and **** *p* < 0.0001 vs. the control, the data were deemed significant.

After reporting the antiproliferative and pro-apoptotic properties of HM in both CRC cell lines HT29 and SW620 (33), an evaluation of potential inhibitory activities of HM on CRC progression was worth investigating. Thus, tested at the same concentrations (50, 100, and 200 μg/mL), HM’s effects were assessed on CRC cell migration and invasion using an automated cell analyzer. Compared to untreated cells, HM significantly decreased HT29 cell migration by 25% (*p* = 0.047) and 40% (*p* = 0.0038) at 50–100 and at 200 μg/mL, respectively (Figure 1A). Surprisingly, the migration rate of mCRC SW620 cells with a cell index of 0.5 was found to be a quarter slower than that of HT29 cells, displaying a cell index of 2.0 (Figure 1A). Unlike HT29 cells, HM significantly reduced SW620 cell migration by 90% (*p* < 0.001) and 70% (*p* = 0.004) at 50–100 and 200 μg/mL, respectively, compared to untreated cells (Figure 1A). Regarding cell invasion, the invasion rate of HT29 cells with a cell index of 3.5 was observed to be higher than that of SW620 cells, displaying a cell index of 1.5 (Figure 1B).

Compared to untreated cells, HM drastically inhibited, in a dose-dependent manner, HT29 cell invasion (−26.4%, *p* = 0.042 at 50 μg/mL; −85.3%, *p* = 0.00041 at 100 μg/mL; −95%, *p* = 0.00003 at 200 μg/mL of HM) and SW620 (−53%, *p* = 0.0039 at 50 μg/mL; −68.75%, *p* = 0.0036 at 100 μg/mL; −95%, *p* = 0.000029 at 200 μg/mL of HM) cell invasion (Figure 1B). The tumorigenic potential of HM was evaluated using an assay for colony formation. Tested at the highest concentrations, HM significantly inhibited, in a dose-dependent manner, the formation of colonies using HT29 (−55%, *p* = 0.0038 at 100 μg/mL; −88%, *p* = 0.00042 at 200 μg/mL of HM) and SW620 (−80%, *p* = 0.00029 at 100 μg/mL; 90%, *p* = 0.00001 at 200 μg/mL of HM) cells (Figure 1C). Epithelial–mesenchymal transition (EMT) protein markers such as epithelial E-cadherin and mesenchymal N-cadherin play a major role in the tumorigenic potential of cancer cells; therefore, their expression levels were monitored in CRC cells treated with 100 μg/mL of HM using Western blotting technology. The E-cadherin expression level significantly increased in HT29 cells treated with HM (1.25-fold, *p* = 0.046) and HM-treated SW620 cells (1.35-fold, *p* = 0.045), while N-cadherin expression level decreased in HT29 cells treated with HM (0.7-fold, *p* = 0.039) and HM-treated SW620 cells (0.35-fold, *p* = 0.001), compared to the control, i.e., untreated cells (Figure 1D). Using RT-qPCR assays, HM-induced E-cadherin upregulation and N-cadherin downregulation were also observed in regard with the expression level of the genes compared to the control (Figure 1E).

### 3.2. HM Downregulates uPAR Expression Level Through JNK and ERK Pathways

**Figure 2:**
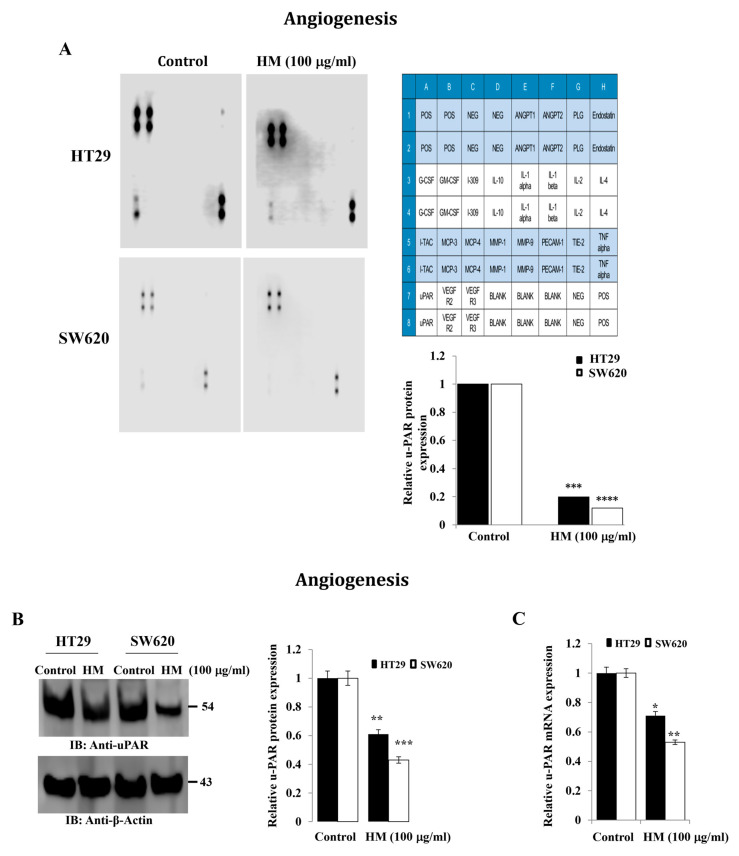
**(A) Analysis of human angiogenesis antibody array in HT29 and SW620 cell lysates after HM treatment.** Whole HT29 and SW620 CRC cell lysates were subjected to immunoblotting against 23 antibodies. In the HT29 and SW620 cell lines, HM downregulated uPAR expression. Bar graphs showing the results expressed in a single experiment (single membrane). (**B**) HT29 and SW620 whole-cell lysates were subjected to immunoblotting against uPAR. The bar graph indicates the relative level of protein expression in relation to the loading control β-actin. (**C**) Bar graph illustrating the relative level of *uPAR* mRNA expression in relation to *GAPDH mRNA,* the internal control. Results from three independent experiments (*n* = 3) are displayed in bar graphs as mean ± SD. When reporting * *p* < 0.05, ** *p* < 0.01, *** *p* < 0.001, and **** *p* < 0.0001 vs. the control, the *p*-values data were deemed significant.

CRC progression, including cell migration and invasion, mainly requires tissue microenvironment remodeling caused by various extracellular proteolysis-related mechanisms, including the uPAR/uPA system. Using a human angiogenesis antibody array for detection of 23 angiogenesis-related proteins, uPAR was the only protein visualized in the lysates of HT29 and SW620 cells after cell exposure in the presence or absence of HM tested at 100 μg/mL. Compared to the control, a significant decrease in uPAR expression level by 0.2-fold (*p* = 0.0009) and 0.15-fold (*p* = 0.000098) was noticed in HT29 and SW620 cells treated with HM, respectively (Figure 2A). Using Western blotting and RT-qPCR assays, HM significantly decreased uPAR protein (−40%, *p* = 0.0042 for HT29; −60%, *p* = 0.001 for SW620) and transcript (−30%, *p* = 0.049 for HT29; −50%, *p* = 0.0046 for SW620) expression levels in both CRC cell lines compared to the control (Figure 2B,C).

**Figure 3:**
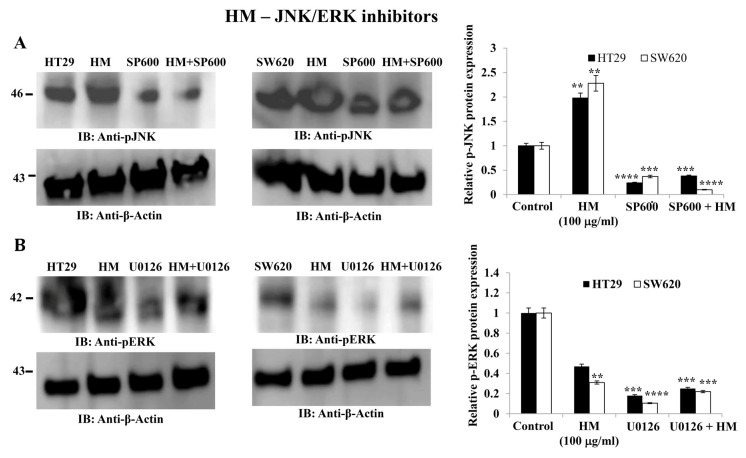
**uPAR expression level in HT29 and SW620 cells after HM treatment following pretreatment with JNK inhibitor SP600125 and MEK inhibitor UO126.** (**A**) HT29 and SW620 whole-cell lysates with/without 10 μM JNK kinase inhibitor (+/− SP600125) were subjected to immunoblotting against pJNK. (**B**) HT29 and SW620 whole-cell lysates with/without 10 μM MEK inhibitor (+/− UO126) were subjected to immunoblotting against pERK. The bar graph shows the relative level of protein expression in relation to the loading control β-actin. Results from three separate experiments (*n* = 3) are displayed in bar graphs as mean ± SD. When reporting ** *p* < 0.01, *** *p* < 0.001, and **** *p* < 0.0001 vs. the control, the data were deemed significant.

To investigate the signaling pathways involved in HM-downregulated uPAR expression, two major oncogenic MAPK pathways, JNK and ERK, widely known to modulate uPAR expression and oncogenic functions [39,40], were studied. Compared to untreated cells, HM significantly increased JNK phosphorylation (2.0-fold, *p* < 0.01, Figure 3A) and decreased ERK phosphorylation (0.4-fold, *p* < 0.01, Figure 3B) in both CRC cell lines. Specific pharmacological inhibitors of JNK and MEK, named SP600125 and UO126, respectively, were tested at the optimal concentration, determined by pilot studies for blocking the phosphorylation of JNK and ERK in both CRC cells. Compared to the significant reduction in JNK phosphorylation levels (~0.3-fold, *p* < 0.001, Figure 3A) and ERK (~0.4-fold, *p* < 0.01, Figure 3B) detected in HT29 and SW620 cells, HM significantly attenuated JNK and ERK phosphorylation blockade caused by both pharmacological inhibitors in the CRC cell lines (Figure 3).

**Figure 4:**
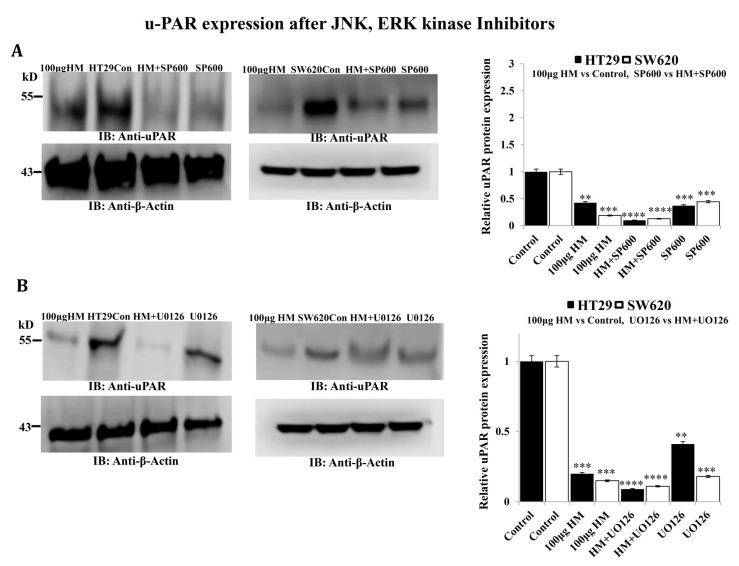
uPAR expression levels in HT29 and SW620 cells after HM treatment following pretreatment with JNK inhibitor SP600125 and MEK inhibitor UO126. (**A**) HT29 and SW620 whole-cell lysates with/without 10 μM JNK kinase inhibitor (+/− SP600125) were subjected to immunoblotting against uPAR. (**B**) HT29 and SW620 whole-cell lysates with/without 10 μM MEK inhibitor (+/− UO126) were subjected to immunoblotting against uPAR. Bar graph showing the relative level of protein expression in relation to the loading control β-actin. Results from three independent experiments (*n* = 3) are displayed in bar graphs as mean ± SD. When reporting ** *p* < 0.01, *** *p* < 0.001, and **** *p* < 0.0001 vs. the control, the data were deemed significant.

Regarding the uPAR protein expression level, JNK pharmacological inhibitor SP600125 significantly decreased uPAR (−0.45-fold, *p* < 0.001) to the same extent as HM tested at 100 μg/mL compared to the control (Figure 4A). While UO126 (0.2-fold, *p* < 0.001) significantly inhibited uPAR to the same extent as HM in SW620 cells, a lesser decrease in uPAR expression level was observed after addition of UO126 (0.4-fold, *p* = 0.0049) compared to the level of uPAR detected in HM-treated HT29 cells (Figure 4B).

**Figure 5:**
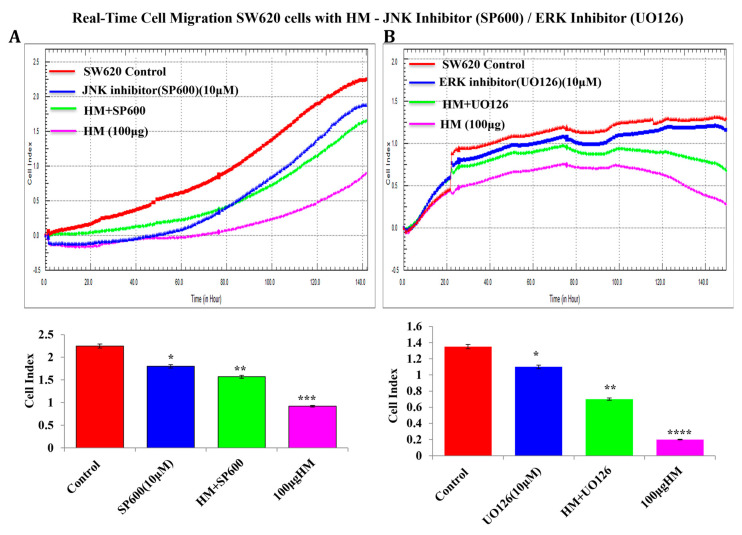
**JNK inhibitor SP600125 (A) and MEK inhibitor UO126 (B) attenuated HM-inhibited real-time SW620 cell migration.** Data are presented as mean ± SD from three separate experiments (*n* = 3). When reporting * *p* < 0.05, ** *p* < 0.01, *** *p* < 0.001, and **** *p* < 0.0001 vs. the control, the data were deemed significant.

At the functional level, focusing on the mCRC cell line SW620, real-time cell migration was recorded after cell exposure to SP600125 and UO126 with or without HM. Compared to the control, both SP600125 and UO126 significantly decreased by 20% (*p* < 0.05) SW620 cell migration (Figure 5). The strongest inhibition of SW620 cell migration caused by HM tested at 100 μg/mL was alleviated after cell pretreatment with SP600125 and UO126 (Figure 5).

## 4. Discussion

The search for new natural anticancer products against CRC progression has attracted more and more intensive research interest [41,42]. Among these studies, HM extracted from the black seed coat of *Nigella sativa* L. exhibits multiple properties with promising beneficial effects in clinical practice, including its antiproliferative effects in colorectal adenocarcinoma and mCRC cell lines via induction of apoptosis, which minimizes inflammation and reduces side effects [34,35]. The main cellular events implicated in CRC progression include cell migration and invasion driven by extracellular/cell surface proteolysis involving the uPA/uPAR system [40]. Sustained uPAR expression has also been associated with tumor angiogenesis, a complex process contributing to tumor spread, drug resistance, and subsequently cancer recurrence. To combat the progression of CRC, the development of various drugs inhibiting or suppressing uPA/uPAR systems has emerged [27,28]. In the present study, HM suppressed uPAR expression, suggesting that HM is a potential uPAR inhibitor for the treatment of CRC among other natural products tested in clinical trials such as polyphenolic phytoalexin resveratrol [43,44]. The investigation of the signaling pathways involved in HM-downregulated uPAR expression revealed, by blocking the JNK and MEK pathways using their specific pharmacological inhibitors, an attenuation of HM-suppressed uPAR expression and HM-inhibited mCRC cell migration. Altogether, this current study demonstrated that HM could inhibit CRC progression by suppressing uPAR expression through JNK and ERK pathways, suggesting that HM is a promising uPAR inhibitor.

Having been extensively studied for its antiproliferative effects in CRC cell lines through apoptosis induction and MAPK signaling modulation via its main receptor TLR4 [34,35], it was crucial to evaluate HM’s effects on CRC progression. To assess the in vitro biological impact of HM on CRC progression, an automated cell analyzer system recording the migration and invasion of colon adenocarcinoma HT29 and metastatic SW620 CRC cell lines was employed. At the various concentrations tested (50, 100, and 200 μg/mL), HM inhibited both CRC progression-driven cellular events (i.e., migration and invasion) and reduced the colony formation assay, which was linked with an induction of epithelial EMT marker E-cadherin expression and a decrease in mesenchymal EMT marker N-cadherin expression levels. These HM antitumor effects described in this current study are consistent with anticancer drugs leading to drastic delay in cancer cell migration, invasion, and colony formation accompanied by reverse expression of EMT proteins, upregulating epithelial markers while decreasing mesenchymal markers [45,46]. Associated signaling pathways were also investigated and revealed the role of AMP-activated protein kinase (AMPK)/nuclear factor (NF)-κB and transcriptional coactivator bearing a PDZ-binding motif (TAZ) in the antitumor activities of natural anticancer drugs [45,46]. In addition to the activation of NF-κB, a downstream effector of TLR4 and which has been demonstrated to mediate HM’s anti-inflammatory effects [47], these signaling pathways, particularly TAZ and AMPK, could also be investigated in terms of the antitumor effects of HM in CRC. Very little research activity has been conducted on HM; however, various types of melanins from different origins, such as bacteria (i.e., *Streptomyces glaucescens*, *Pseudomonas strains*) and microneedle patches, have exhibited anticancer activities in HFB4 skin cancer cells and in mice bearing melanoma and breast carcinoma [48,49]. Furthermore, HM and natural melanins are oligomeric nano-aggregate polymers with interesting physical properties, such as photoreceptors and electron transfer agents, presenting photo-protective and radio-protective potentials, suggesting a promising beneficial impact of melanin for cancer prevention and treatment in clinical practice.

Cancer progression, which exhibits dramatic features changes, including high cell motility, surrounding tissue invasion, tumor microenvironment adaptation, survival in bloodstream, and chemoresistance, occurs mainly through extracellular/cellular proteolysis, principally mediated by the uPA/uPAR system [50]. Thus, uPAR becomes a crucial therapeutic anticancer target leading to the development of uPAR inhibitors, including engineered drugs, synthetic, or even natural compounds [27,28]. In this current study, HM suppressed uPAR expression through ERK and JNK MAPK pathways in CRC cell lines HT29 and SW620. These MAPK pathways have been demonstrated to be involved in transcription factor activator protein 1 (AP1)-mediated uPAR gene expression in metastatic cancer [39,51]. A wide variety of natural anticancer compounds have emerged as suppressors of uPAR expression, such as resveratrol through ERK1/2 downstream effector of EGFR, docosahexaenoic acid (DHA) via heme oxygenase-1, and ursolic and oleanolic acids in a matrix metalloproteinase (MMP)-dependent pathway [52,53,54]. Recently, an albumin-based drug carrier targeting uPAR was reported to show promising anticancer progression in hepatoma H22-bearing mice [55]. Furthermore, it has been mentioned that melanin and melanin-functionalized nanoparticles exhibit beneficial anticancer properties with radioprotective effects [36]. Through this current study, the demonstration of suppression of uPAR gene expression by HM may open new avenues for the development of HM-based drug carriers targeting uPAR, which could improve the treatment modalities against CRC progression.

## 5. Conclusions

Our findings disclose the anti-CRC activities of HM, evidenced by an inhibition of CRC cell migration and invasion, upregulation of E-cadherin, and downregulation of N-cadherin and associated with a decrease in uPAR expression. Anti-oncogenic HM added to CRC cells pretreated with JNK and MEK inhibitors attenuated the blockade of JNK and ERK phosphorylation and alleviated HM-downregulated uPAR expression and HM-inhibited mCRC cell migration. Overall, our in vitro studies demonstrate that HM exhibits an inhibitory effect on mCRC migration and invasiveness, associated with uPAR downregulation through JNK and ERK pathways, suggesting HM as a promising uPAR inhibitor against invasive cancer cells. While more in-depth mechanistic in vitro investigations are warranted, further in vivo experiments, including xenografts and models of metastasis, are needed to validate HM’s anti-metastatic effects in CRC.

## Figures and Tables

**Table 1:** Primer sequences.

Target Genes	Primer Sequences
*E-cadherin*	F: 5′-ACCAGAATAAAGACCAAGTGACCA-3′
	R: 5′-AGCAAGAGCAGCAGAATCAGAAT-3′
*N-cadherin*	F: 5′-ATTGGACCATCACTCGGCTTA-3′
	R: 5′-CACACTGGCAAACCTTCACG-3′
*uPAR*	F: 5′-TGCAATGCCGCTATCCTACA-3′
	R: 5′-TGGGCATCCGGGAAGACT-3′
*GAPDH*	F: 5′-AAGGTCGG AGTCAACGGATTTGGT-3′
	R: 5′-ATGGCATGGACTGTGGTCATAGT-3′

## Data Availability

The data presented in this study are available on request from the corresponding author.
